# Severe multivessel coronary artery spasm detected by computed tomography: a case report

**DOI:** 10.1093/ehjcr/ytaa369

**Published:** 2020-11-22

**Authors:** Cai De Jin, Moo Hyun Kim, Su-A Jo, Kyunghee Lim

**Affiliations:** 1 Department of Cardiology, Dong-A University Hospital, 26 Daesingongwon-ro, Seo-gu, Busan 49201, Republic of Korea; 2 Department of Cardiology, Affiliated Hospital of Zunyi Medical University, Zunyi 563003, Guizhou, China

**Keywords:** Coronary computed tomography angiography, Multivessel coronary artery spasm, Detect, Case report

## Abstract

**Background:**

Ventricular arrhythmia and sudden cardiac arrest caused by multivessel coronary artery spasm (CAS) is rare. Although coronary angiography (CAG) with provocation testing is the diagnostic gold standard in current vasospastic angina guidelines, it can cause severe procedure-related complications. Here, we report a novel technique involving dual-acquisition coronary computed tomography angiography (CCTA) to detect multivessel CAS in a patient who survived out-of-hospital cardiac arrest (OHCA).

**Case summary:**

A 58-year-old healthy Korean male survived OHCA caused by ventricular fibrillation (VF), experiencing seven episodes of defibrillation and cardiopulmonary resuscitation, and was referred to the Emergency Room. Vital signs were stable and physical examination, electrocardiogram, chest, and brain CT did not show any abnormal findings, except elevated hs-Troponin I levels (0.1146 ng/mL). Echocardiogram revealed a regional wall motion abnormality in the inferior wall, with a low normal left ventricular ejection fraction (50%). A multivessel CAS (both left and right) was detected using a dual-acquisition CCTA technique (presence and absence of intravenous nitrate). During CAG with the 2^nd^ injection of ergonovine, a prolonged and refractory total occlusion in the proximal-ostial right coronary artery was completely relieved after a seven-cycle intracoronary injection regimen of nitroglycerine. The patient was discharged with the recommendation of smoking and alcohol cessation. Nitrate and calcium channel blockers were also prescribed. The patient had no further events at 3 months of follow-up after discharge.

**Discussion:**

Dual-acquisition CCTA is a promising tool to detect multivessel CAS.

**Learning points**

- Severe multivessel specific coronary artery spasm (CAS) likely initiates unpredictable ventricular fibrillation-out-of-hospital cardiac arrest.
- Dual-acquisition coronary computed tomography angiography is a novel tool for the detection of multivessel CAS.

## Introduction

Coronary artery spasm (CAS) can cause predominant rest angina and temporary ischaemia with transient ischaemic ST-segment deviation that promptly responds to sublingual nitrates [(named vasospastic angina (VSA)], which is caused by abnormal spontaneous or induced constriction of coronary vessels. In rare cases, multivessel CAS can lead to life-threatening ventricular arrhythmias or sudden cardiac arrest.^1^^,^^2^ Although invasive coronary angiography (CAG) with provocation testing is the diagnostic gold standard in current VSA guidelines,^3^ it may potentially lead to ergonovine-related life-threatening complications,^4^ and it is also difficult to observe both the left and right coronary artery simultaneously. As a non-invasive method of cardiac imaging, to avoid these complications is difficult with coronary computed tomography angiography (CCTA), but it can reduce vascular access site complications, while provide additional information. Previously we proposed CCTA as a tool for the detection of CAS in clinical practice.^5^^,^^6^ Here, we present a case of survival following ventricular fibrillation (VF)-related OHCA due to severe multivessel CAS detected by dual-acquisition CCTA in a middle-aged healthy man who was diagnosed with VSA.

## Timeline

**Table:** 

**21 March 2020 04:30–04:58**	Chest discomfort, cardiac arrest caused by ventricular fibrillation, successful resuscitation witd cardiopulmonary resuscitation and defibrillation.
**21 March 2020 07:09**	Referred to Dong-A University Hospital. Physical examination, electrocardiogram, chest and brain computed tomography (CT) observations were unremarkable.
**23 March 2020**	Echocardiogram revealed a regional (inferior) wall motion abnormality, with a low normal left ventricular ejection fraction (50%). No ventricular arrhythmias or abnormal findings during Holter monitoring.
**24 March 2020**	No specific abnormal findings on abdominal ultrasonography.
**25 March 2020 07:37**	Coronary computed tomography angiography (CCTA) found diffused, moderate stenosis in multivessel coronary arteries. Focal myocardial thinning with perfusion defect hypokinesia in the basal inferoseptal segment within the right coronary artery (RCA).
**25 March 2020**	Coronary angiography with provocation testing induced a total occlusion in the proximal RCA, which was completely relieved after a seven-cycle administration of intracoronary nitroglycerine.
**25 March 2020 10:31**	A second acquisition CCTA with the intravenous nitrate technique according to dual-acquisition CCTA protocol-derived criteria.
**26 March 2020**	The patient was discharged with smoking and alcohol cessation. Nitrate and calcium channel blockers were prescribed.
**10 July 2020**	No further complaints during follow-up.

## Case presentation

**Figure 1:**
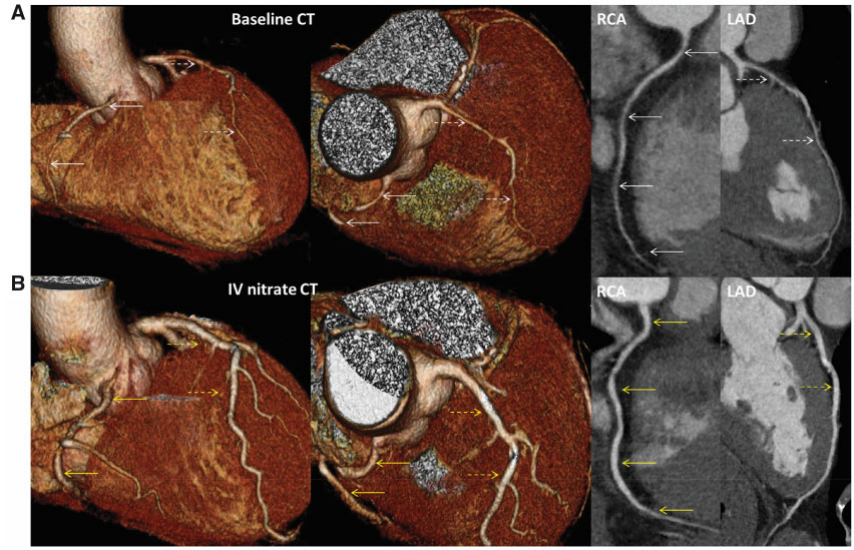
Three-dimensional constructed computed tomography imaging acquisition of the heart on volume rendering and curved multiplanar reformation view. (*A*) Baseline computed tomography reveals diffuse luminal narrowing of multivessel coronary arteries with invisible side branches (especially in left anterior descending artery and right coronary artery). (*B*) IV nitrate computed tomography showed that the lumen and vessel of the coronary arteries were larger than those at baseline computed tomography, and side branches had emerged again. White arrows indicate initial spastic right coronary artery (solid) and left anterior descending artery (dotted); Yellow arrows indicate nitrate-relieved right coronary artery (solid) and left anterior descending artery (dotted). CT, computed tomography; IV, intravenous; LAD, left anterior descending artery; RCA, right coronary artery.

**Figure 2:**
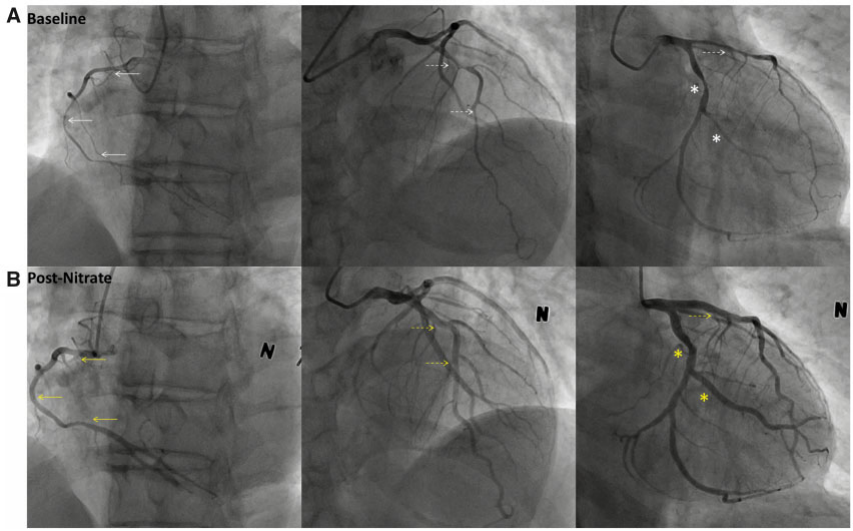
Coronary angiograms after RCA-first ergonovine provocation testing in a survivor of OHCA. (A) Baseline RCA and LCA angiograms, diffuse and multifocal narrowing vessel in RCA and LCA (LAD-diagonals, LCX-obtuse marginal bifurcation presenting triangle-shaped imaging features). (B) The RCA flow and vessel were totally relieved until seven cycles of IC nitroglycerine (200 μg for each cycle in 10∼20-second intervals), and LCA with side branch (both LAD-diagonals and LCX-obtuse marginal) vessels recovered to a normal size, in the absence of triangle-shaped imaging features. Although the patient experienced intolerable chest pain during the provocation test procedure, fortunately, there was no occurrence of life-threatening ventricular arrhythmia or cardiac arrest. RCA videos are available online in the Supplementary Data. White arrows indicate initial spastic RCA (solid), LAD (dotted) and LCX (star); Yellow arrows indicate nitrate-relieved RCA (solid), LAD (dotted) and LCX (star).

A 58-year-old healthy Korean male who was a heavy drinker and smoker survived OHCA caused by VF in the early morning and was referred to Dong-A University Emergency Room (ER). The patient had no past medical history. The emergency condition (OHCA) developed just minutes after reports of chest discomfort. When emergency medical services arrived at his home, he was found to be in VF and was successfully resuscitated after seven cycles of cardiopulmonary resuscitation and defibrillation. Subsequently, he was transferred to the ER of our hospital for further management. His vital signs were stable, and physical examination, electrocardiogram (normal sinus rhythm), chest, and brain CT did not reveal any abnormal findings with the exception of elevated hs-Troponin I levels (0.1146 ng/mL) (normal <0.0342 ng/mL). Echocardiogram revealed a regional (inferior) wall motion abnormality with low normal left ventricular ejection fraction (50%). After 4 days of coronary care unit observation, the patient exhibited stable hemodynamics as well as no ventricular arrhythmias or abnormal findings including ischaemic or long QT changes, we decide to do coronary angiogram and spasm provocation as well as CCTA as a research protocol. Coronary computed tomography angiography imaging revealed moderate luminal stenosis in the left anterior descending artery (LAD), left circumflex artery (LCX), and right coronary artery (RCA), appearing as a spasm-like coronary phenomenon (*Figure 1A*). A focal myocardial thinning with perfusion defect was also detected, consistent with an ischaemic finding in the RCA territory. Subsequent CAG findings were multifocal, diffusely narrowed lumen in the RCA, and triangle-shaped imaging features in LAD-diagonals and LCx-obtuse marginal bifurcation. A total occlusion in the proximal region, near the ostium of the RCA after the 2^nd^ dose of ergonovine administration was observed (10 μg at 1-min intervals). Right coronary artery spasm was refractory to intracoronary (IC) administration of nitroglycerine, and the patient complained prolonged intolerable chest pain with ST elevation in the inferior leads. Additional sublingual nitroglycerine and repeated IC nitroglycerine were administered, until the seventh cycle of IC nitroglycerine injection was reached (200 μg for each cycle in 10∼20-second intervals). RCA flow was finally restored after several minutes of refractory pain. After the nitrate injections, LCA with side branch vessels returned to a normal size and the triangle-shaped imaging features simultaneously disappeared (*Figure 2*, online video links are available in the Supplementary material online). Fortunately, the patient did not experience life-threatening ventricular arrhythmia or cardiac arrest throughout the entire ergonovine provocation test procedure. The subsequent second acquisition CCTA with intravenous (IV) nitrate confirmed the compatible finding of multivessel CAS (especially in LAD and RCA, shown in *Figure 1B*) according to dual-acquisition CCTA protocol-derived criteria.^5^ The patient was discharged with the recommendation of smoking and alcohol cessation, and nitrate and calcium channel blockers were prescribed. The patient reported no further events at 3 months of follow-up after discharge.

## Discussion

Sudden cardiac arrest often arises from acute coronary syndrome, or other structural heart diseases (arrhythmogenic right ventricular cardiomyopathy and hypertrophy cardiomyopathy), cardiac channelopathy (long QT syndrome, catecholaminergic polymorphic ventricular tachycardia, and Brugada syndrome), as well as pulmonary embolism, aortic dissection and intracranial haemorrhage.^7^

Although CAS can lead to life-threatening complications including lethal arrhythmias or sudden cardiac arrest and was initially thought to be a rare event,^8^ recent reports suggest that CAS accounts for 7% of OHCA cases.^9^ Lee *et al*.,^10^ reported that some patients experience recurrent events, which rise in probability across the 4 years after the initial episode of coronary vasospasm despite medical treatment. In 2019, Yasue *et al*.,^11^ reported that patients with multivessel CAS were more likely to suffer from sudden cardiac arrest. Due to these findings and based on the Japanese Coronary Spasm Association (JCSA) risk-stratification score system, our patient was considered in the high-risk strata, with an approximate 3.1% risk of recurrent hard major adverse cardiovascular events, including cardiac death, non-fatal myocardial infarction and implantable cardioverter defibrillator (ICD) shocks.^12^ The prognosis of VSA patients with aborted sudden cardiac arrest is often poorer. Thus, ICD in high-risk patients could be seen as a secondary preventive measure because current multiple vasodilator therapy appears to be less ideal.^13^ In addition, ICD combined with calcium channel blockers is the current preferred approach for preventing recurrence.^12^^,^^14^ In this case, the cardiologist had fully considered the higher recurrence of VF-OHCA, but the patient declined ICD implantation despite a strong recommendation, and thus only received conservative management with combined anti-anginal drugs, together with the recommendations of cessation of smoking and alcohol as well as avoidance of triggers (i.e. hyperventilation, cold, exercise, magnesium deficiency, and cocaine).

Dual-acquisition CCTA as a novel technique for the detection of CAS was first initiated at Dong-A University’s Cardiovascular Center and appears to be particularly useful for multivessel spasms. The CT scan protocol and previous findings have been detailed previously.^5^^,^^6^ The technique can be applied in the presence and absence of nitrate during dual-acquisition CCTA and represents a non-invasive alternative to invasive imaging for CAS detection. The NAVIGATOR study (Clinical Trial Registration: URL: https://www.clinicaltrials.gov. Unique identifier: NCT03570671) is currently underway to provide valuable information in regards to diagnostic accuracy in CAS, and to support the creation of CCTA-based diagnostic criteria for patients with VSA. The patient who was the subject of this case report was eligible and enrolled in the NAVIGATOR study.

## Conclusion

In a survivor of VF-OHCA induced by severe multivessel CAS, dual-acquisition CCTA in the presence and absence of IV nitrate aided in the detection of multivessel CAS.

## Lead author biography

**Figure:**
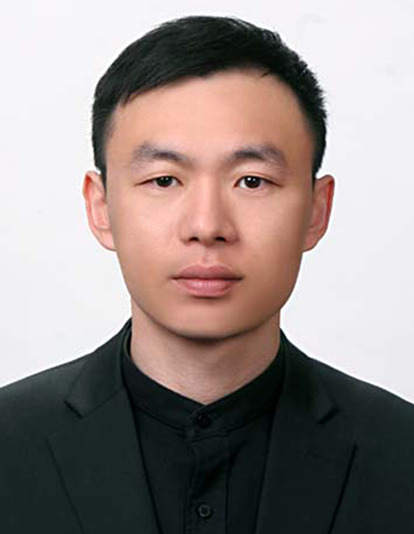


Dr Cai De Jin was born in Wangqing, Yanbian Korean Autonomous Prefecture, Jilin, China. He is a physician in department of Cardiology (Affiliated Hospital of Zunyi Medical University), who received his doctoral degree in Medicine in 2016 and completed his post-doctoral work in 2017 from Dong-A University (Republic of Korea), and finished Fellowship of coronary interventional training under the Professor Moo Hyun Kim at Dong-A University Hospital. His interest is mainly focused on platelet research in acute coronary syndrome, cardiac imaging modalities, and coronary intervention.

## Supplementary material

Supplementary material is available at *European Heart Journal* - *Case Reports* online.

## Supplementary Material

ytaa369_Supplementary_Data
