# Supplementary material for: Severe multivessel coronary artery spasm detected by computed tomography: a case report
Source: Eur Heart J Case Rep. 2020 Nov 22;4(6):1–5. doi: 10.1093/ehjcr/ytaa369 (PMC7793155; doi:10.1093/ehjcr/ytaa369)
Supplement: ytaa369_Supplementary_Data [file ytaa369_supplementary_data.zip › ytaa369-suppl_data/EHJ-CR Spasm revise R2.pptx]

## Slide 1
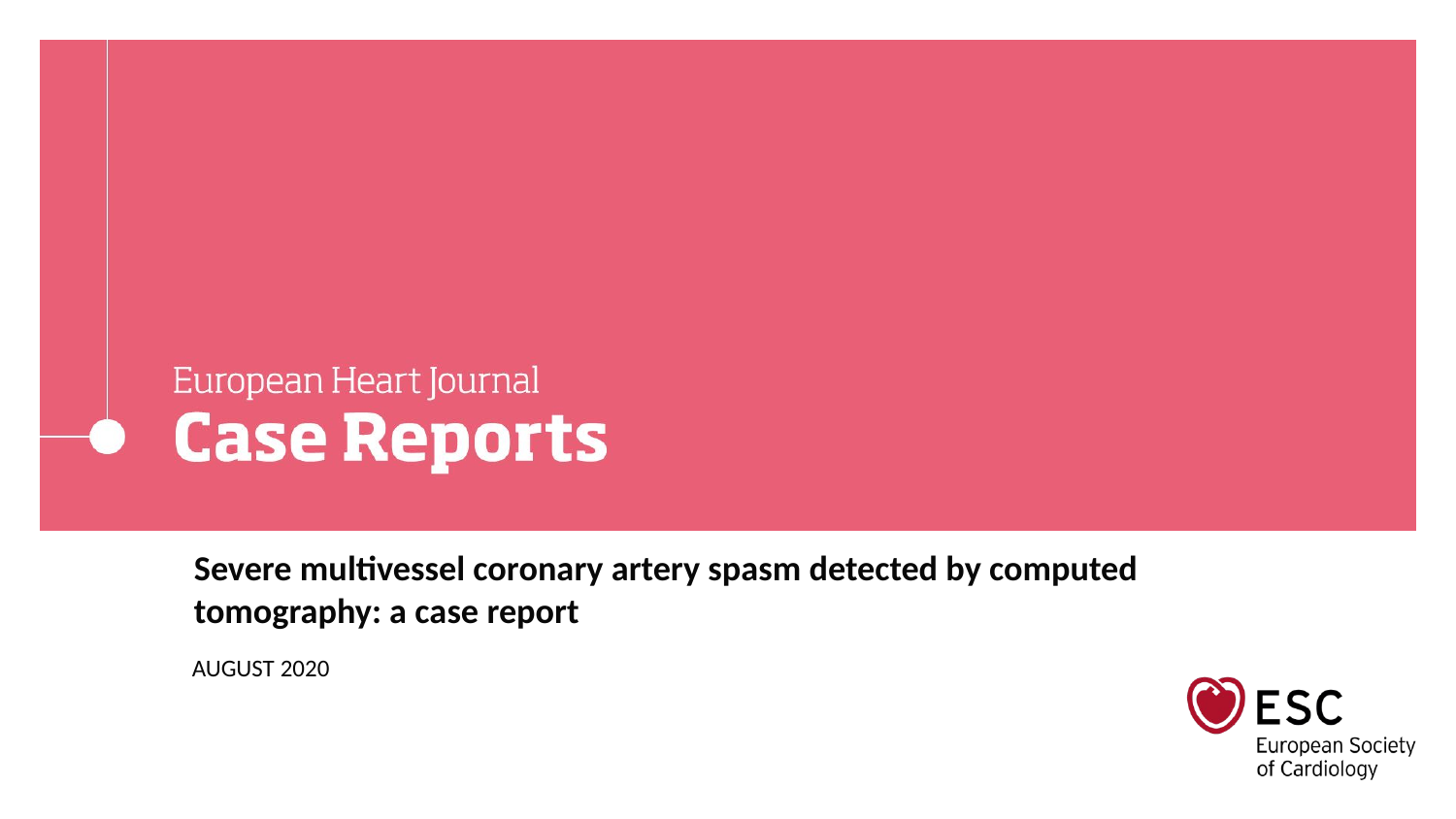

# Severe multivessel coronary artery spasm detected by computed tomography: a case report
AUGUST 2020

## Slide 2
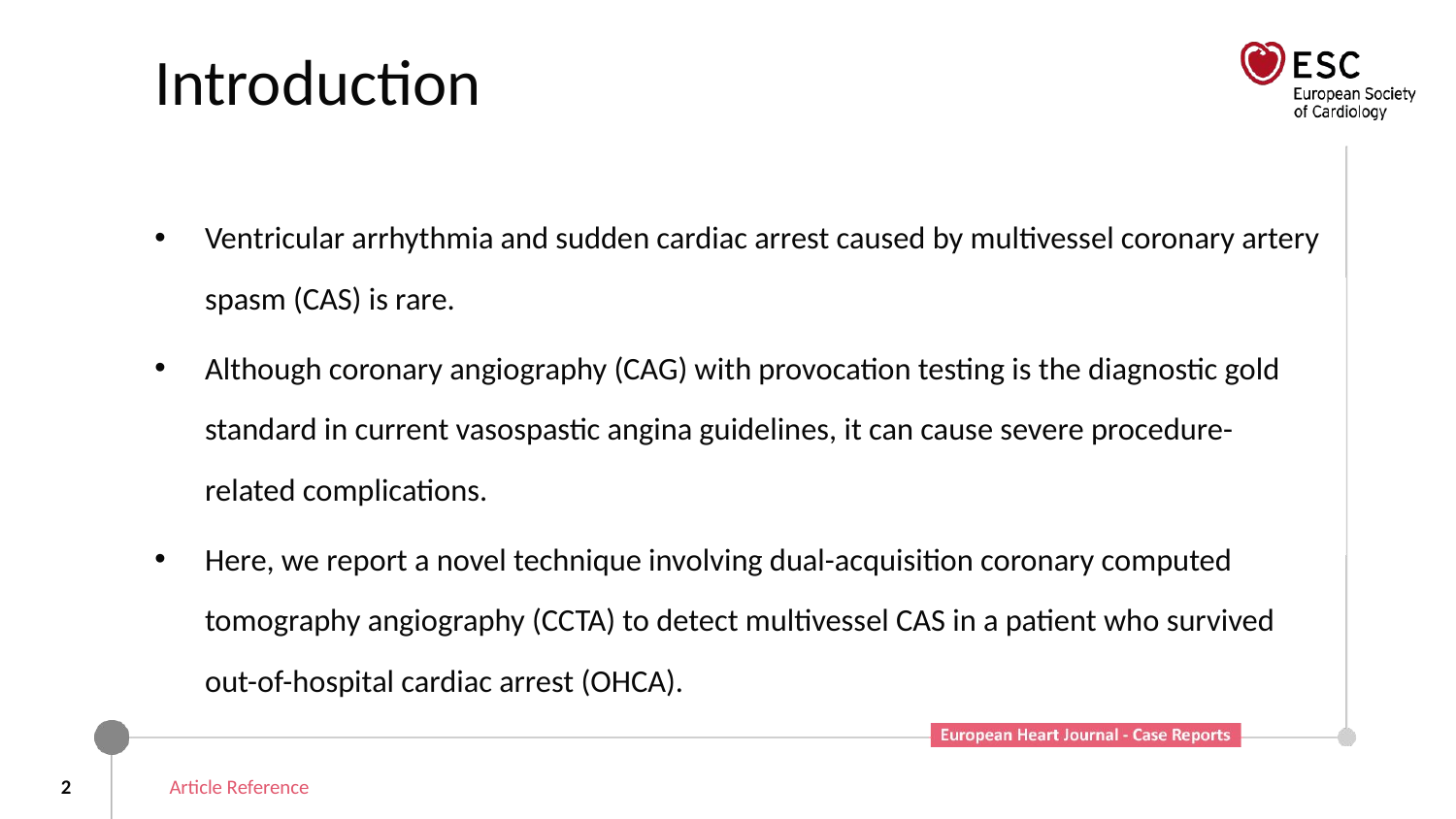

# Introduction
Ventricular arrhythmia and sudden cardiac arrest caused by multivessel coronary artery spasm (CAS) is rare.
Although coronary angiography (CAG) with provocation testing is the diagnostic gold standard in current vasospastic angina guidelines, it can cause severe procedure-related complications.
Here, we report a novel technique involving dual-acquisition coronary computed tomography angiography (CCTA) to detect multivessel CAS in a patient who survived out-of-hospital cardiac arrest (OHCA).
2
Article Reference

## Slide 3
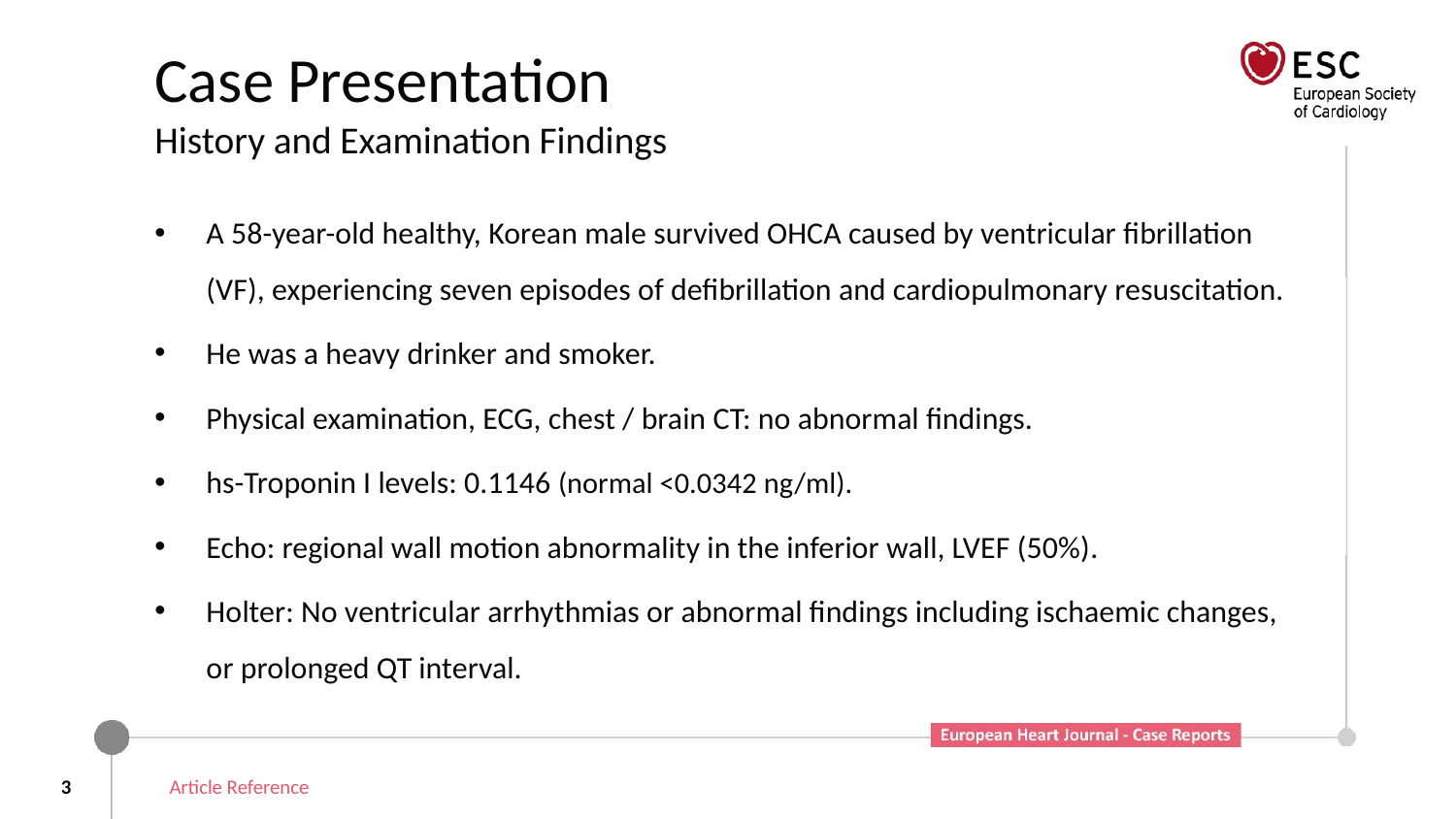

# Case Presentation History and Examination Findings
A 58-year-old healthy, Korean male survived OHCA caused by ventricular fibrillation (VF), experiencing seven episodes of defibrillation and cardiopulmonary resuscitation.
He was a heavy drinker and smoker.
Physical examination, ECG, chest / brain CT: no abnormal findings.
hs-Troponin I levels: 0.1146 (normal <0.0342 ng/ml).
Echo: regional wall motion abnormality in the inferior wall, LVEF (50%).
Holter: No ventricular arrhythmias or abnormal findings including ischaemic changes, or prolonged QT interval.
3
Article Reference

## Slide 4
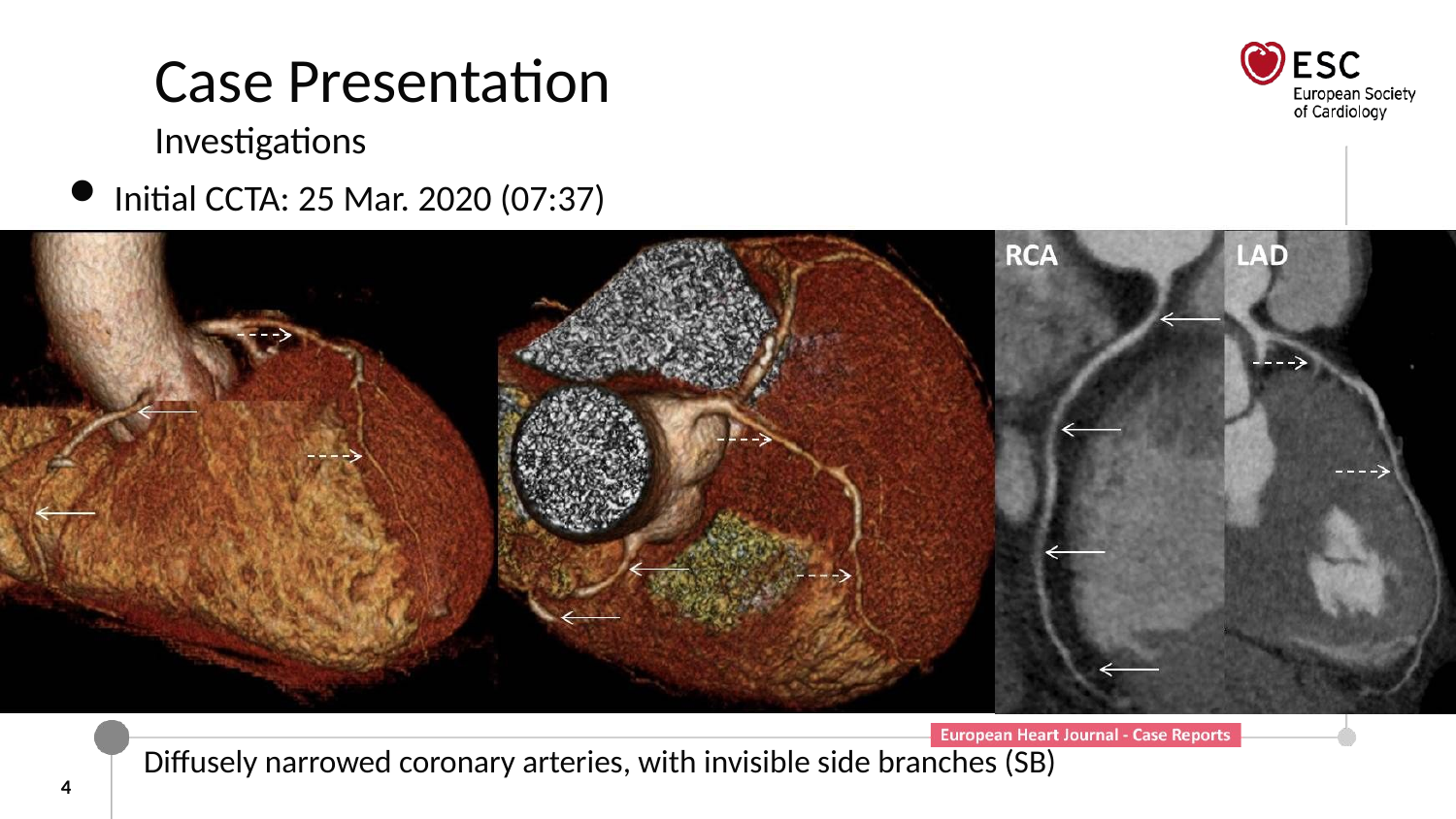

# Case Presentation Investigations
Initial CCTA: 25 Mar. 2020 (07:37)
Diffusely narrowed coronary arteries, with invisible side branches (SB)
4

## Slide 5
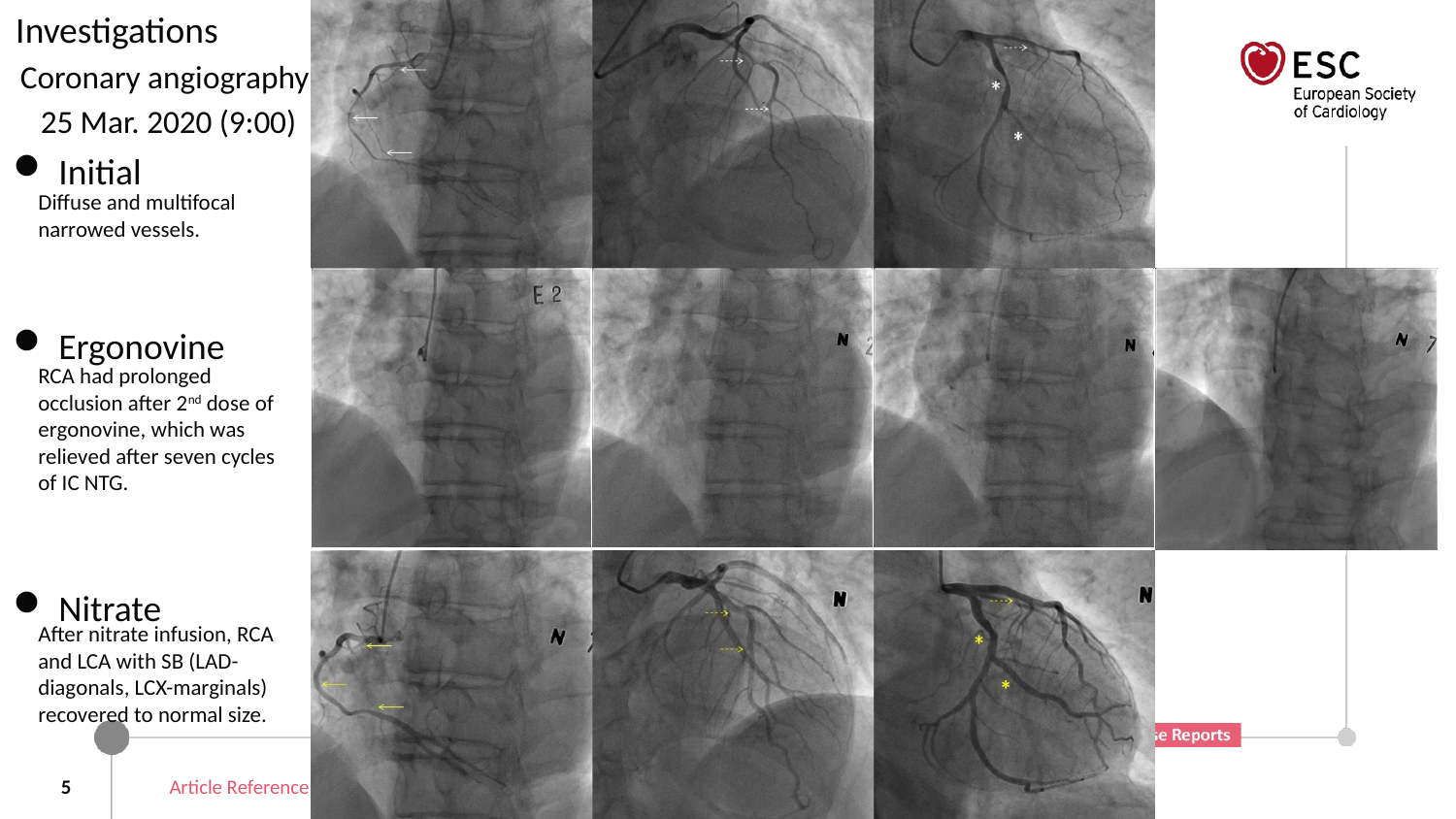

Investigations
Coronary angiography
25 Mar. 2020 (9:00)
Initial
Ergonovine
Nitrate
Diffuse and multifocal narrowed vessels.
RCA had prolonged occlusion after 2nd dose of ergonovine, which was relieved after seven cycles of IC NTG.
After nitrate infusion, RCA and LCA with SB (LAD-diagonals, LCX-marginals) recovered to normal size.
5
Article Reference

## Slide 6
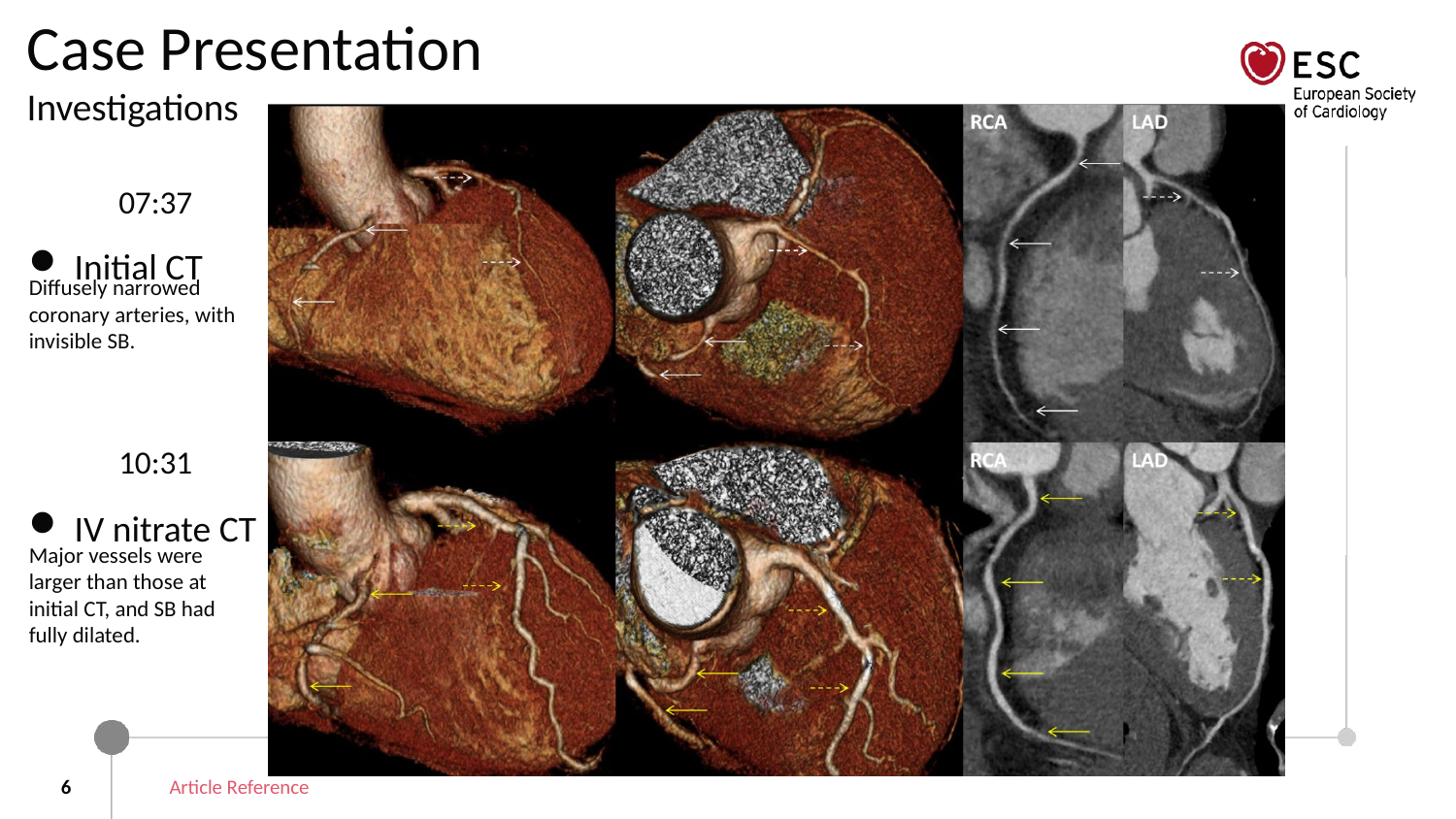

# Case Presentation Investigations
Initial CT
IV nitrate CT
07:37
Diffusely narrowed coronary arteries, with invisible SB.
10:31
Major vessels were larger than those at initial CT, and SB had fully dilated.
6
Article Reference

## Slide 7
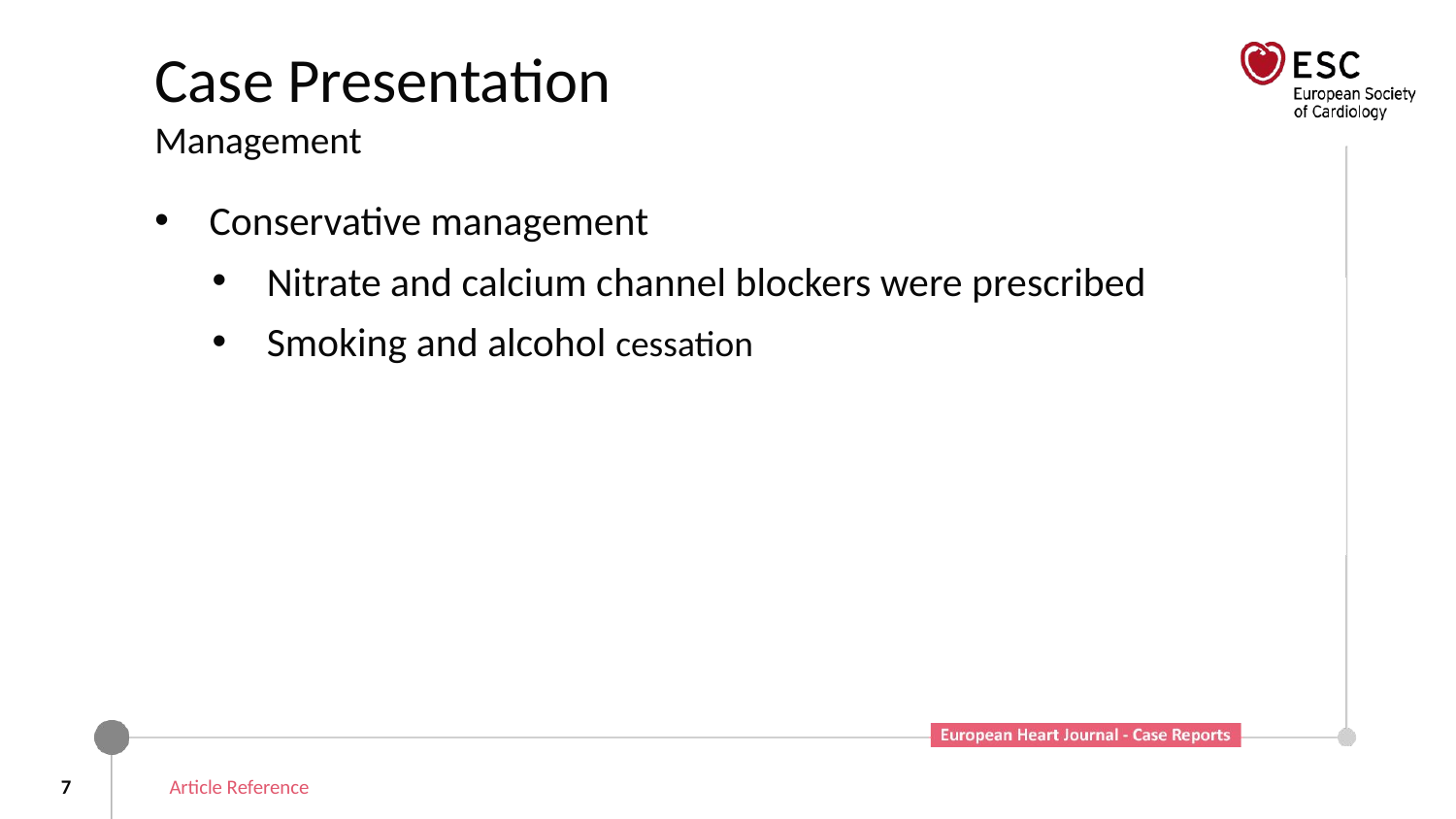

# Case Presentation Management
Conservative management
Nitrate and calcium channel blockers were prescribed
Smoking and alcohol cessation
7
Article Reference

## Slide 8
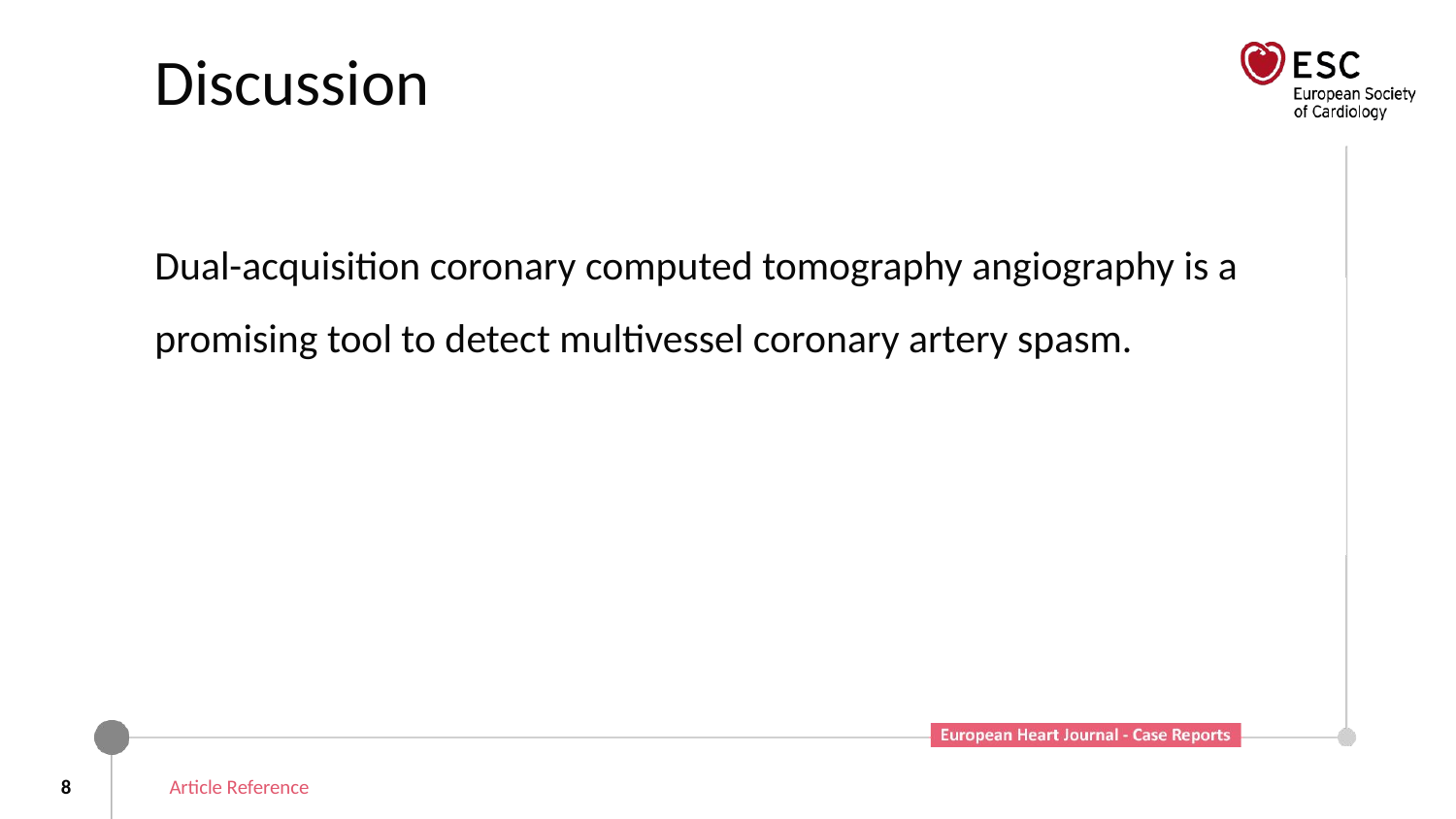

# Discussion
Dual-acquisition coronary computed tomography angiography is a promising tool to detect multivessel coronary artery spasm.
8
Article Reference

## Slide 9
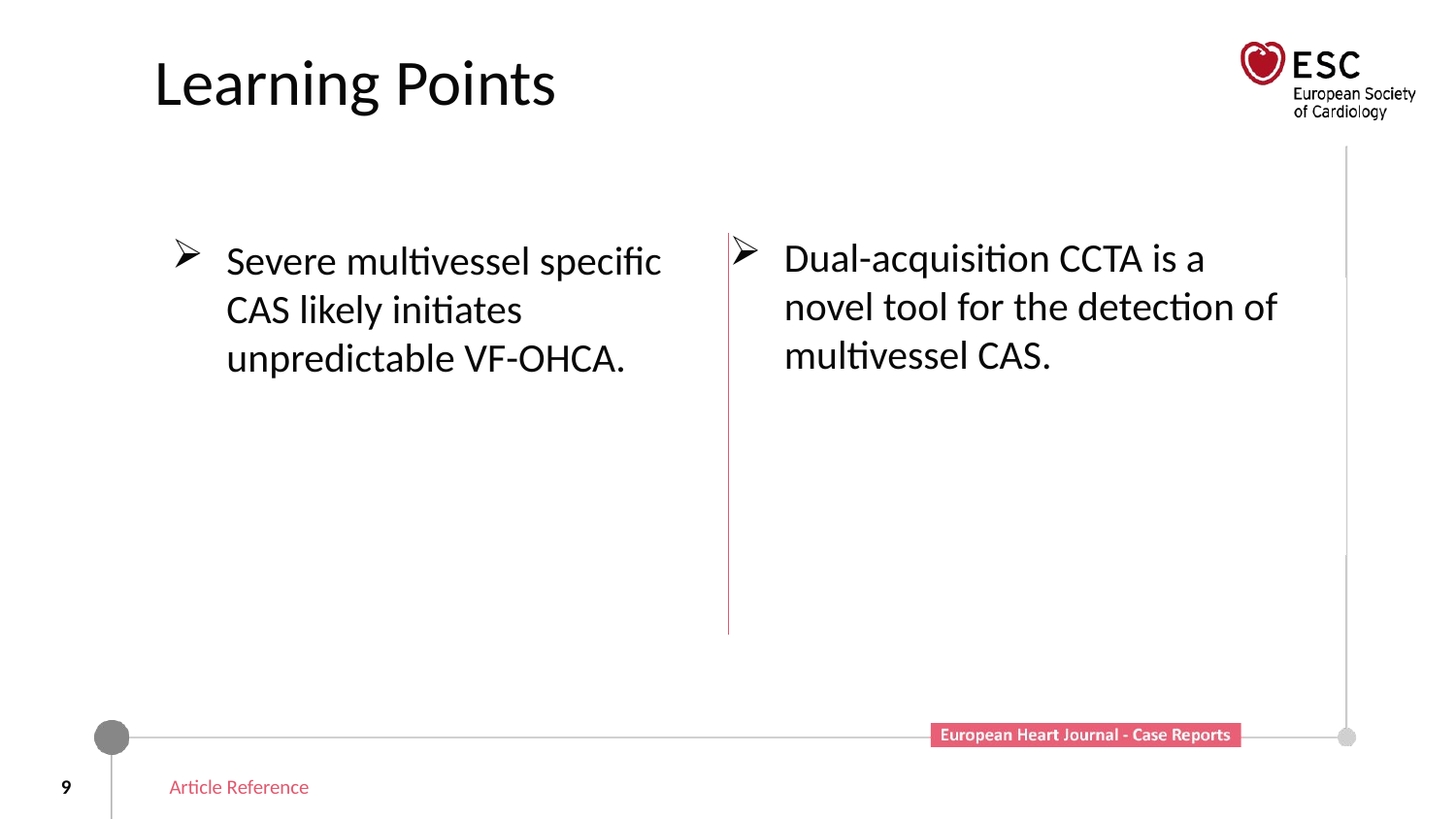

# Learning Points
Dual-acquisition CCTA is a novel tool for the detection of multivessel CAS.
Severe multivessel specific CAS likely initiates unpredictable VF-OHCA.
9
Article Reference

## Slide 10
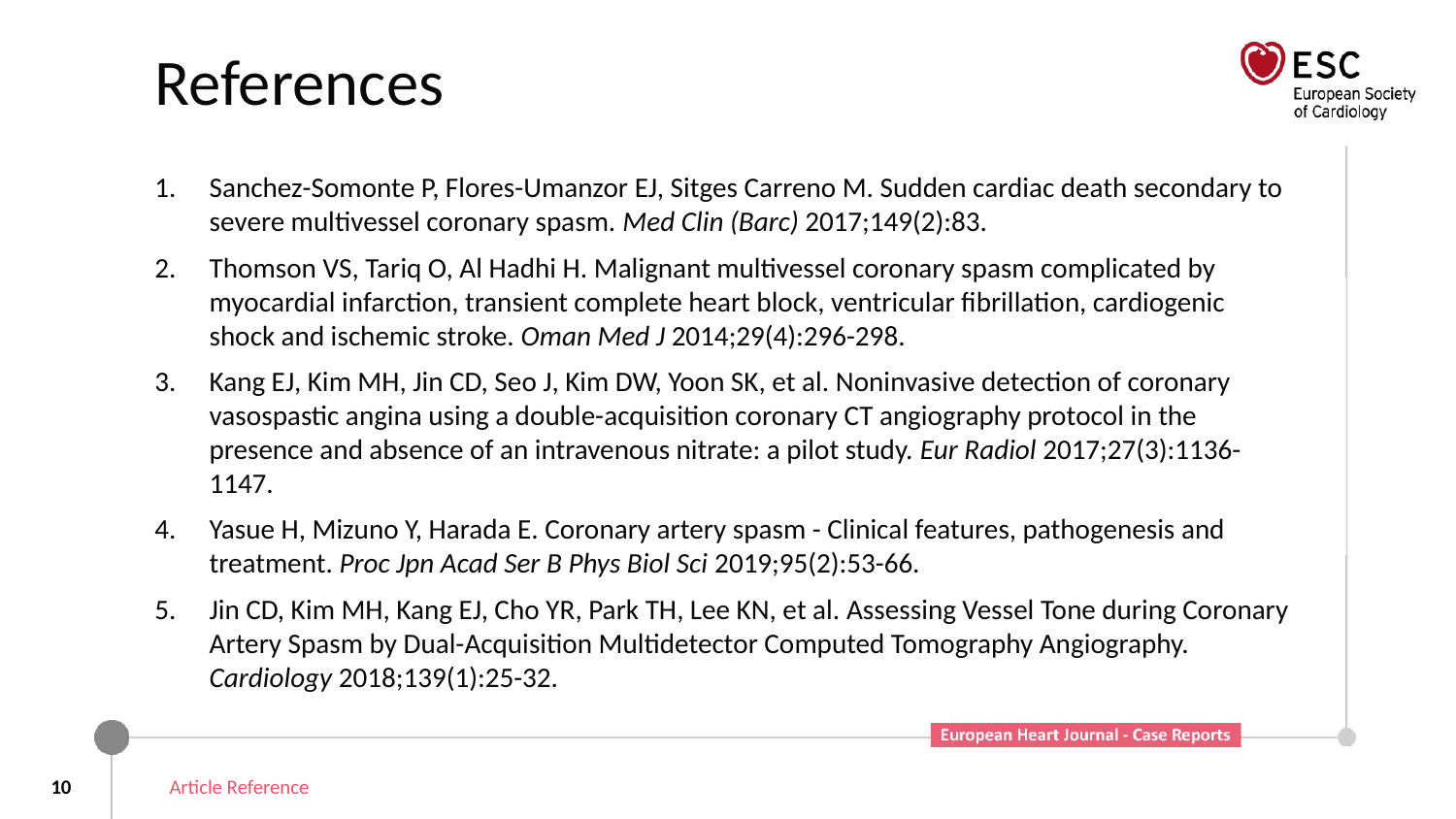

# References
Sanchez-Somonte P, Flores-Umanzor EJ, Sitges Carreno M. Sudden cardiac death secondary to severe multivessel coronary spasm. Med Clin (Barc) 2017;149(2):83.
Thomson VS, Tariq O, Al Hadhi H. Malignant multivessel coronary spasm complicated by myocardial infarction, transient complete heart block, ventricular fibrillation, cardiogenic shock and ischemic stroke. Oman Med J 2014;29(4):296-298.
Kang EJ, Kim MH, Jin CD, Seo J, Kim DW, Yoon SK, et al. Noninvasive detection of coronary vasospastic angina using a double-acquisition coronary CT angiography protocol in the presence and absence of an intravenous nitrate: a pilot study. Eur Radiol 2017;27(3):1136-1147.
Yasue H, Mizuno Y, Harada E. Coronary artery spasm - Clinical features, pathogenesis and treatment. Proc Jpn Acad Ser B Phys Biol Sci 2019;95(2):53-66.
Jin CD, Kim MH, Kang EJ, Cho YR, Park TH, Lee KN, et al. Assessing Vessel Tone during Coronary Artery Spasm by Dual-Acquisition Multidetector Computed Tomography Angiography. Cardiology 2018;139(1):25-32.
10
Article Reference
